# Is *OPRM1* genotype a valuable predictor of VAS in patients undergoing laparoscopic radical resection of colorectal cancer with fentanyl?

**DOI:** 10.1186/s12871-023-02120-1

**Published:** 2023-05-22

**Authors:** Yan Zhou, Lei Cao, Yihui Yang, Yaoyi Gao, Yihao Li, Beili Wang, Baishen Pan, Jian Huang, Wei Guo

**Affiliations:** 1 Department of Laboratory Medicine, Zhongshan Hospital, Fudan University, 111 Yi Xue Yuan Road, Shanghai, 200032 People’s Republic of China; 2 Department of Pharmacy, Zhongshan Hospital, Fudan University, 111 Yi Xue Yuan Road, Shanghai, 200032 People’s Republic of China; 3 Department of Anesthesiology, Zhongshan Hospital, Fudan University, 111 Yi Xue Yuan Road, Shanghai, 200032 People’s Republic of China

**Keywords:** *OPRM1* A118G, PACU VAS, Risk factors, Fentanyl dose

## Abstract

**Objective:**

This study was conducted to examine the association between the A118G polymorphism of the *OPRM1* gene and the risk of increased VAS scores in patients with colorectal cancer who underwent laparoscopic radical resection for which fentanyl was used.

**Methods:**

The *OPRM1* A118G genotype in subjects were detected. The relationship between the A118G polymorphism of the *OPRM1* gene and increased Visual Analogue Scale (VAS) scores throughout the perioperative period was explored. A total of 101 patients receiving fentanyl anesthesia undergoing laparoscopic radical resection of colon tumors at Zhongshan Hospital, Fudan University between July 2018 and December 2020 were investigated in the present study. The relative risk between the A118G polymorphism of the *OPRM1* gene and VAS ≥ 4 in the PACU was estimated using the adjusted effect relationship diagram, baseline characteristic analysis, and multiple logistic regression analysis. The relationship between the A118G polymorphism of the *OPRM1* gene and VAS in the PACU, as well as perioperative fentanyl usage, was examined after confounders were adjusted.

**Results:**

Subjects with *OPRM1* A118G wild gene A were less sensitive to fentanyl, which was a risk factor for PACU VAS ≥ 4. Before the model was adjusted, the odds ratio (OR) was 14.73 (*P* = 0.001). After adjusting for age, sex, weight, height, and the duration of surgery, the OR increased to 16.55 (*P* = 0.001). When adjusting for age, sex, weight, height, surgery duration, *COMT* Val158Met gene polymorphism, CYP3A4 *1G gene polymorphism, and CYP3A5 *3gene polymorphism, the OR was 19.94 (*P* = 0.002). Moreover, *OPRM1* A118G wild type gene A was found to be a risk factor for increased dosage of fentanyl in the PACU. Before the model was adjusted, the OR reached 16.90 (*P* = 0.0132). After adjusting for age, sex, body weight, intraoperative fentanyl dosage, surgery duration, and height, the OR was 13.81, (*P* = 0.0438). When adjusting for age, sex, weight, height, intraoperative fentanyl dosage, surgery duration, *COMT* Val158Met gene polymorphism, *CYP3A4* *1G gene polymorphism, and *CYP3A5* *3 gene polymorphism, the OR reached 15.23, (*P* = 0.0205).

**Conclusion:**

The A118G polymorphism of the *OPRM1* gene carrying wild gene A was a risk factor for VAS ≥ 4 in the PACU. Moreover, it is a risk factor for increased dosage of fentanyl in the PACU.

**Supplementary Information:**

The online version contains supplementary material available at 10.1186/s12871-023-02120-1.

Effective pain management following surgery is critical to accelerating postoperative recovery, shortening the hospitalization period, and improving patients’ satisfaction [1]. At present, fentanyl is widely used in anesthesia and patient-controlled analgesia (PCA) because of its strong and quick analgesic effect [2]. Its usage and dose are mainly based on the patients’ height and weight, as well as the clinical experience of the anesthesiologist. However, the response to fentanyl varies among people. Insufficient or excessive dose is prone to occur during the anesthesia process. The former will result in insufficient analgesia, while the latter may result in severe respiratory depression [3, 4]. Individualized application of fentanyl is critical to perioperative pain management and clinical treatment.

Many studies have shown that individual differences in fentanyl response are closely related to human genetics. Mu-opioid receptor (MOR) is the main target of fentanyl and is encoded by the human opioid receptor-1 gene (*OPRM1*). One of its SNPs, named A118G, is prevalent and crucial in *OPRM1,* which results in an amino acid change in the extracellular glycosylation site of MOR cells.*OPRM1* A118G polymorphism can affect the function of MOR [5, 6]. On the other hand, studies have shown that gene polymorphisms of CYP3A4*1G, *COMT* Val158Met and *CYP3A5* *3 are closely related to the analgesic effect of opioids.

In this study, we investigated the relationship between *OPRM1*, *CYP3A4*, *COMT*, and *CYP3A5* gene polymorphisms and perioperative VAS. We aimed to assess whether these gene polymorphisms were associated with an increased risk of VAS.

## Materials and methods

### Study subjects

This research included 101 patients who underwent laparoscopic resection for colon cancer between July 2018 and December 2020. All included patients were informed and signed informed consent.

*Inclusion criteria*: patients aged 45 to 70 years old; patients receiving laparoscopic radical resection with periumbilical incision where in vitro anastomosis was done; patients’ physiological status before surgery classed as American Society of Anesthesiologists (ASA) class I-II.

*Exclusion criteria*: intraoperative switch from a laparoscopic surgery to laparotomy; intracorporeal anastomosis following laparoscopic surgery; patients with morbid obesity or extreme emaciation (BMI > 30 or < 20 kg/m^2^), liver and kidney dysfunction, and chronic pain; patients who have taken enzyme inducers and inhibitors that affect CYP3A4 enzyme activity within 1 month.

*Withdrawal criteria*: subjects asked to withdraw informed consent, change in operation mode during operation, the intraoperative blood loss reaching more than 500 ml.

Data were obtained from patient medical records. The following variables were considered in our modeling: age, weight, height, intraoperative fentanyl dose, operation time, gender, and genotype. Other variables, such as comorbidity/ASA, and tumor location (right/left) were supplied in Supplementary Material 1 (S1).

### Analgesia methods and evaluation

No preoperative medication was applied to patients before they entered the operating room. The anesthesia mask was used for supplying oxygen and removal nitrogen before induction. Fentanyl 3 μg/kg, propofol 2 mg/kg, CIS atracurium 0.2 mg/kg were used for intravenous induction. No supplementary respiration was performed during the induction. Endotracheal intubation was performed three minutes after all intravenous induction was completed. Spontaneous respiration was cultivated after peritoneal suture. The dose of fentanyl was adjusted according to the spontaneous respiratory rate. After the patients regained consciousness, the tracheal tube was removed and they were sent to the postanesthesia care unit (PACU).

Patient-controlled intravenous analgesia (PCIA) was performed with the PCA pump. The pump was attached to the intravenous tube after the tracheal tube was removed. The patient was observed in PACU for one hour. If the visual analogue scale/score (VAS) ≥ 4, the patient-controlled dose of fentanyl, i.e. 20 μg, was delivered. If necessary, it could be given repeatedly until the VAS was less than 4. The analgesic effect and adverse reactions were observed in PACU.

### Evaluation indexes and methods

Blood pressure, heart rate, pulse, oxygen saturation, Electrocardiogram, and other vital indicators of the patients were all monitored. The following domains were recorded: the fentanyl consumption; and the VAS from the beginning of surgery to 48 h after surgery.

The primary endpoint was that the spontaneous breathing rate of patients was stable at ≤ 10 times/minute before the removal of the tracheal tube, and the secondary endpoint was VAS of less than 4 when the patient left PACU.

### Genotyping

Main instruments and reagents included Applied Biosystems 7500 Fast Realtime PCR System (Thermo Fisher Scientific (China) Co., Ltd.), AB3730 sequencer (ABI, USA), Lab-Aid 820 Midi reagent for nucleic acid extraction (Xiamen Zhishan Biotechnology Co., Ltd.), Permix Taq@Hot Start Version (Bao Bioengineering (Dalian) Co., Ltd.), and sequencing reagent (Thermo Fisher Scientific (China) Co., Ltd.).

Two milliliters of EDTA-K2 anticoagulant were collected before operation. Lab-Aid 820 Midi reagent was used for DNA extraction in strict accordance with the instructions. The DNA was then stored at -20℃ until detected. The software Oligo7 was used for PCR primer design, as shown in Table 1

**Table 1:** The primer of genes

Gene Name and SNP Loci	Primer Name	Primer Sequence (5’––3’)
CYP3A4*1G *rs2242480*	rs2242480-F	CAAGGAACACACCCATAACACT
	rs2242480-R	TAGAAAGCAGATGAACCAGAGC
CYP3A5*3 *rs776746*	rs776746-F	GTCCTTGTGAGCACTTGATG
	rs776746-R	AGCCCGATTCTGCAGCTGGA
*COMT* Val158Met *rs4680*	rs4680-F	ACAGGCAAGATCGTGGACGC
	rs4680-R	CACCTTGGCAGTTTACCCAG
*OPRM1* A118G *rs563649*	rs*563649*-F	TTGGACTTTAAATATGGCAA
	rs*563649*-R	CATACATTGGAAATACTTAG

Reaction conditions: 94 ℃ for 5 min; 45 cycles (94 ℃ for 30 s; 61 ℃ for 30 s; 72 ℃ for 30 s); 72 ℃ for 5 min; insulation at 4 ℃. After purification, the PCR amplification products were sequenced on ABI 3730 DNA Analyzer, and the results were analyzed by the Chromas software.

The present study conformed to the principles of the Declaration of Helsinki. Approval was obtained from the Research Ethics Committee of the Zhongshan Hospital, Fudan University (Approval number: B2018-054R).

### Statistical analysis

Data are expressed as mean (SD) for continuous variables and percentage (%) for categorical variables. The continuous variables included age, weight, height, intraoperative fentanyl dose, and operation time. The categorical variables included gender, and genotype. The dose–response curve was plotted with the curve-fitting method. The multivariable regression model included additional variables such as age, weight, height, the intraoperative dose of fentanyl, operation time, gender, and *OPRM1*, *CYP3A4*, *COMT*, and *CYP3A5* polymorphism. R version 3.4.1 and the software IBM SPSS (version 21.0) were used to perform statistical analysis. All* P* values for statistics were 2-tailed, and *P* < 0.05 was regarded as statistically significant.

## Results

### Clinical characteristics

**Fig. 1:**
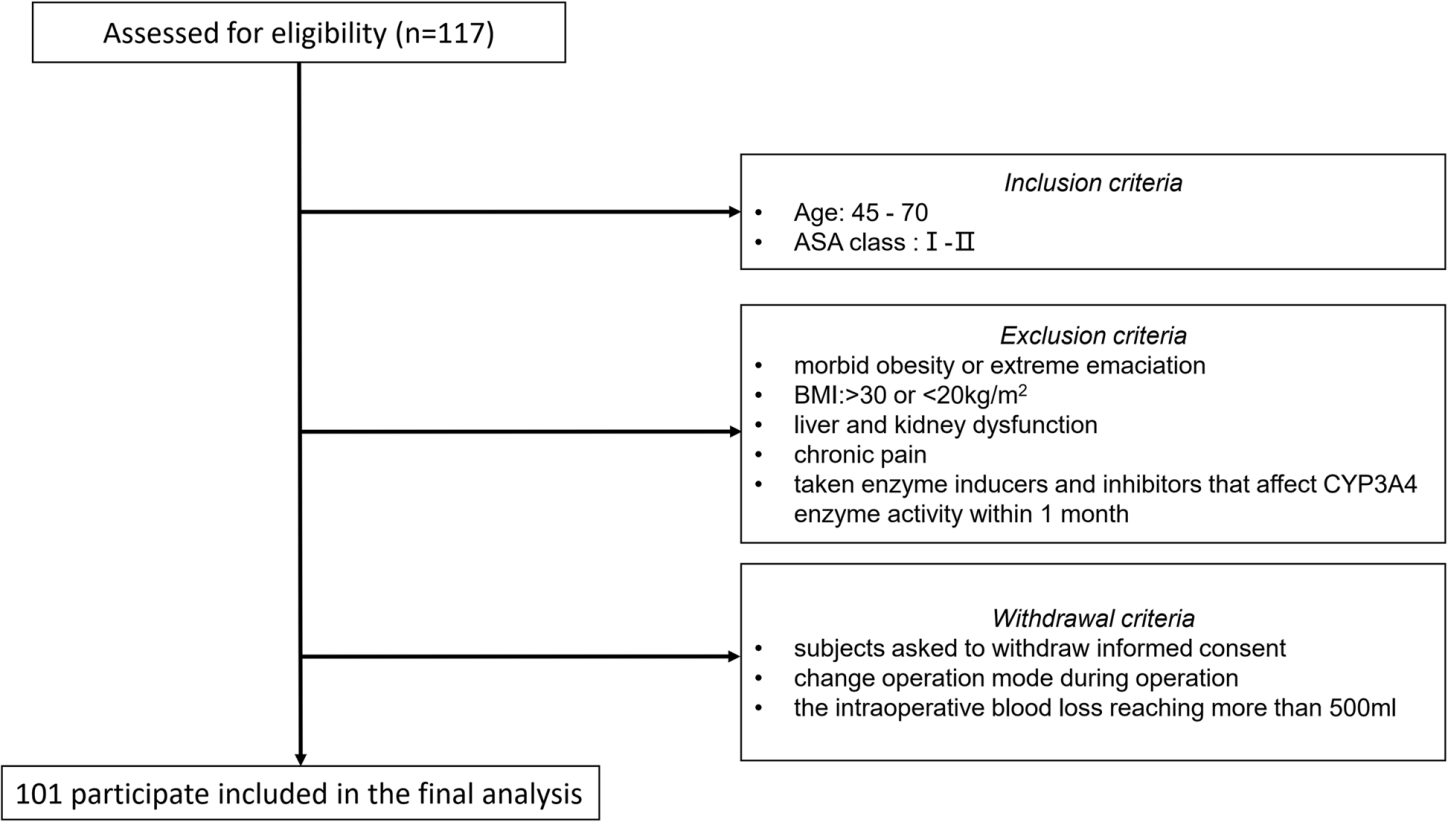
The chart of the participants

Figure 1 contains the flow chart of the participants. Table 2 describes the baseline characteristics of the subjects, including demographic characteristics and some laboratory test results that may be related to assess the VAS. The clinical and outcome data for subjects were obtained from clinical records. Table 2 describes all patient data, including age, gender, weight, height, fentanyl dosage, operation time, and genotypes of *OPRM1* A118G, *CYP3A4* *1G, *CYP3A5* *3, and *COMT* Val158Met.

**Table 2:** Characteristics of subjects in the research

Characteristic	VAS < 4	VAS ≥ 4
NO of paticipants	92	9
Age	59.53 ± 5.87	61.67 ± 4.00
Gender		
Male	53 (57.61%)	5 (55.56%)
Female	39 (42.39%)	4 (44.44%)
Weight	62.23 ± 9.17	64.22 ± 7.60
Height	164.80 ± 7.77	162.89 ± 6.53
Dosage of fentanyl	8.95 ± 2.02	10.21 ± 2.28
Operation time	2.33 ± 0.54	2.26 ± 0.33
*CYP3A4* *1G		
*1/*1	47 (51.09%)	3 (33.33%)
*1/*1G + *1G/*1G	45 (48.91%)	6 (66.67%)
*OPRM1* A118G		
GG	81 (88.04%)	4 (44.44%)
GA + AA	11 (11.96%)	5 (55.56%)
*COMT* Val158Met		
GG	48 (52.17%)	5 (55.56%)
GA + AA	44 (47.83%)	4 (44.44%)
*CYP3A5* *3		
GG	43 (46.74%)	2 (22.22%)
GA + AA	49 (53.26%)	7 (77.78%)

There was no significant difference in age (59.53 ± 5.87 vs 61.67 ± 4.00), gender, weight (62.23 ± 9.17 vs 64.22 ± 7.60 kg), height (164.80 ± 7.77 vs 162.89 ± 6.53 cm), fentanyl dosage (8.95 ± 2.02 vs 10.21 ± 2.28 μg/kg), operation time (2.33 ± 0.54 vs 2.26 ± 0.33 h), *CYP3A4* *1G, *CYP3A5* *3 or *COMT* Val158Met between VAS < 4 and VAS ≥ 4, as shown in Table 2. There was no significant difference in the diameter of tumor (3.99 ± 1.96 vs 3.48 ± 2.12 cm),comorbidity, ASA(I vs II), and tumor location (right/left), as shown in S1.

Among 101 subjects, the number of VAS ≥ 4 and VAS < 4 was significantly different. There was significant difference in *OPRM1* genotypes between the two groups. Patients carrying wild gene A had decreased fentanyl sensitivity and were more prone to VAS ≥ 4.

### The impact of assessing PACU VAS

The feature selection factors with significant differences in univariate analysis were included in the regression analysis. Based on univariate analysis, multivariate logistic analysis was performed for each factor, including age, sex, weight, height, intraoperative fentanyl dosage, operation duration, *OPRM1* A118G、*CYP3A4* *1G、*CYP3A5* *3 and *COMT* Val158Met polymorphisms, as shown in Table 3.

**Table 3:** Risk factors of VAS

Intercept and variable	Odds ratio	95% CI of OR
Age	1.07	0.94–1.22
Gender		
Male	Reference	
Female	1.09	0.27–4.31
Weight	1.02	0.95–1.11
Height	0.97	0.88–1.06
Dosage of fentanyl	1.30	0.96–1.76
Operation time	0.76	0.19–3.02
*CYP3A4* *1G		
*1/*1	Reference	
*1/*1G + *1G/*1G	2.09	0.49–8.86
*OPRM1* A118G		
GG	Reference	
GA + AA	14.73	3.21–67.49
*COMT* Val158Met		
GG	Reference	
GA + AA	0.87	0.22–3.46
*CYP3A5* *3		
GG	Reference	
GA + AA	0.43	0.08–2.34

Carrying wild gene A was associated with VAS ≥ 4, according to the *OPRM1* A118G polymorphism, and was a risk factor for VAS ≥ 4. Further regression analysis showed that without adjusting of confounders, the VAS ≥ 4 in GA and AA-type subjects was 13.73 times higher than that of GG-type subjects. After fully adjusting the confounding factors that may affect PACU VAS, such as age, sex, weight, height, and operation duration, carrying gene A of *OPRM1* is a risk factor in VAS ≥ 4. Subjects carrying gene A of *OPRM1* were 15.55 times higher than GG-type subjects. After adjusting of age, sex, weight, height, operation duration, and the polymorphisms of CYP3A4*1G, CYP3A5*3, and *COMT* Val158Met, the probability of VAS ≥ 4 in carrying gene A of *OPRM1* subjects was 18.94 times higher than that in GG type subjects, as shown in Table 4.

**Table 4:** Individual effect of *OPRM1* A118G on VAS of PACU

Exposure	Non-adjusted		Adjust model I		Adjust model II	
	**OR(95%CI)**	***P* Value**	**OR(95%CI)**	***P* Value**	**OR(95%CI)**	***P* Value**
***OPRM1* A118G**						
** GG**	**1.0**		**1.0**		**1.0**	
** GA + AA**	**14.73 (3.21, 67.49)**	**0.001**	**16.55 (3.01, 91.15)**	**0.001**	**19.94 (3.14, 126.82)**	**0.002**

Adjustment model I: adjusted according to age, sex, weight, height, and operation duration

Adjustment model II: adjusted according to age, sex, weight, height, operation duration and the polymorphisms of *CYP3A4* *1G、*CYP3A5* *3 and *COMT* Val158Met polymorphisms

**Fig. 2:**
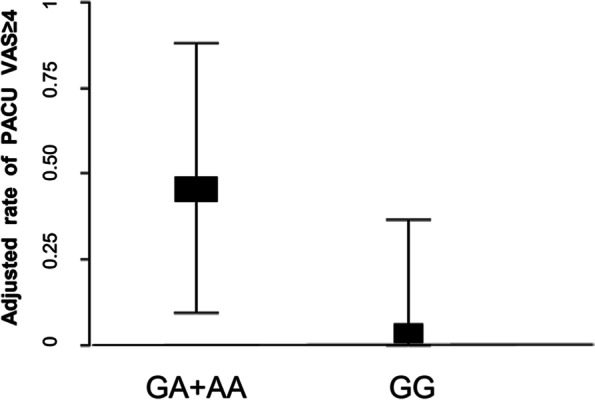
Adjusted effect relationship diagram between OPRM1 A118G and PACU VAS≥4. Adjusted according to age, sex, weight, height, intraoperative fentanyl dosage, operation duration and the polymorphisms of CYP3A4 *1G、*CYP3A5* *3 and *COMT* Val158Met polymorphisms, the risk of PACU VAS ≥ 4 in the subjects carrying gene A of* OPRM1 *A118G is significantly increased

Figure 2 is a diagram of the adjusted effect relationship, which clearly shows that after sequentially adjusting for age, sex, weight, height, intraoperative fentanyl dosage, operation duration and the polymorphisms of *CYP3A4* *1G、*CYP3A5* *3 and *COMT* Val158Met, the probability of PACU VAS ≥ 4 in patients carrying gene A of *OPRM1* is significantly increased.

Adjusted according to age, sex, weight, height, intraoperative fentanyl dosage, operation duration and the polymorphisms of *CYP3A4* *1G, *CYP3A5* *3 and *COMT* Val158Met polymorphisms, the risk of PACU VAS ≥ 4 in the subjects carrying gene A of *OPRM1* A118G is significantly increased. In the GG group, the adjusted mean was 0.036,(95%CI: 0.002–0.366); in the GA + AA group, the adjusted mean was 0.468,(95%CI: 0.096–0.879).

### Independent effect of *OPRM1* A118G gene polymorphism on fentanyl dose in PACU

The polymorphism of *OPRM1* A118G was associated with fentanyl dosage during PACU. Carrying A gene is a risk factor for decreased fentanyl sensitivity and increased fentanyl dosage during PACU. Further regression analysis showed that the probability of GA and AA-type subjects receiving high-dose fentanyl was 15.90 times higher than that of GG-type subjects; After adjusting for age, sex, weight, height, intraoperative fentanyl dosage and operation duration, the probability of GA and AA-type subjects was 12.81 times higher than that of GG-type; After fully adjusting for age, sex, weight, height, intraoperative fentanyl dosage, operation duration, and the polymorphisms of *CYP3A4* *1G, *CYP3A5* *3, *COMT* Val158Met, the probability of subjects carrying A gene was 14.23 folds higher than that of GG homozygous, as shown in Table 5.

**Table 5:** Independent effect of *OPRM1* A118G polymorphism on fentanyl dose in PACU

Exposure	Non-adjusted		Adjust model I		Adjust model II	
	**OR(95%CI)**	***P* Value**	**OR(95%CI)**	***P* Value**	**OR(95%CI)**	***P* Value**
***OPRM1* A118G**						
** GG**	**1.0**		**1.0**		**1.0**	
** GA + AA**	**16.90 (3.78,30.03)**	**0.0132**	**13.81 (0.57,27.06)**	**0.0438**	**15.23(2.57, 27.89)**	**0.0205**

Adjustment model I: adjusted according to age, sex, weight, height, intraoperative fentanyl dosage and operation duration

Adjustment model II: adjusted according to age, sex, weight, height, intraoperative fentanyl dosage, operation duration and the polymorphisms of *CYP3A4* *1G、*CYP3A5* *3, *COMT* Val158Met polymorphisms

**Fig. 3:**
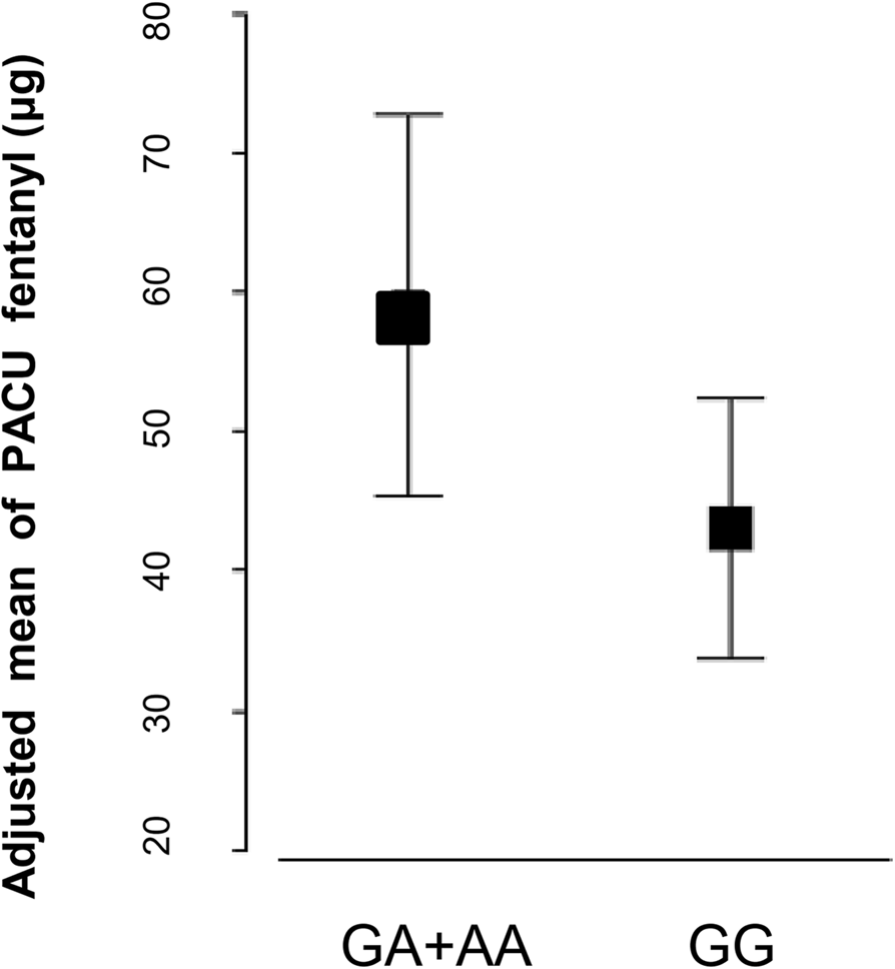
Adjusted effect relationship diagram between *OPRM1* A118G and PACU fentanyl. Adjusted according to age, sex, weight, height, intraoperative fentanyl dosage, operation duration and the polymorphisms of *CYP3A4* *1G、*CYP3A5* *3 and *COMT* Val158Met polymorphisms

The adjusted effect relationship diagram in Fig. 3 clearly shows that after adjusting for age, sex, weight, height, intraoperative fentanyl dosage, operation duration, and the polymorphisms of *CYP3A4* *1G, *CYP3A5* *3, and *COMT* Val158Met, the dose of fentanyl in patients carrying gene A of *OPRM1* is significantly increased in PACU. In the GG group, the adjusted mean was 43.10 μg(95%CI: 33.82–52.37); in the GA + AA group, the adjusted mean was 59.01 μg(95%CI: 45.36–72.65).

## Discussion

With the advancement of the gene and the in-depth investigation of anesthetic medications, more and more studies have revealed that gene polymorphism would result in varying effects of the same anesthetic agent on different populations and individuals [7]. Gene polymorphisms among different populations and individuals cause differences in drug transporters, target receptors, and metabolic enzyme functions, thus affecting the effects of anesthetic drugs. We selected essential genes involved in the mechanism of action and metabolism of fentanyl for our investigation. From the perspective of single nucleotide polymorphism, we discussed the influence of genotype on anesthetic effect, tried to provide a basis for clinical implementation of precision anesthesia. And it may be possible to use drugs rationally according to the characteristics of patients' different genomes, which may improve efficacy, reduce toxicity and prevent adverse reactions.

Fentanyl is a potent synthetic analgesic with an analgesic mechanism similar to morphine [8]. Fentanyl is 60 to 80 times more potent than morphine as an opioid receptor agonist. Compared with morphine and pethidine, it takes effect in a shorter time and does not release histamine. It has little impact on cardiovascular function and can inhibit the stress response during endotracheal intubation.

The VAS scale was employed in this study to assess the pain level of patients during operation [9]. VAS is a simple and effective pain assessment method that may objectively and accurately assess pain. Using the VAS scale can reduce bias caused by exaggerating or ignoring factors, and thus increase pain assessment accuracy. Our study revealed that the *OPRM1* genotype differed significantly between populations with VAS ≥ 4 and VAS < 4. The subjects carrying gene A are less sensitive to fentanyl and more prone to VAS ≥ 4.

The analgesic efficacy and adverse reactions of opioids mainly act on three opioid receptors known as δ, κ, and μ. Among them, μ opioid receptors are the key targets of endogenous and exogenous opioid analgesia, tolerance, dependence and other effects, which are coded by the μ Receptor gene (*OPRM1*). *OPRM1* A118G mutation is the most common and reported functional gene. Some studies have shown that its gene mutation reduces the efficacy of various opioids, and patients with GG need more opioids to relieve pain [10–13].On the other hand, some studies have shown that *OPRM1* A118G mutation can enhance the analgesic effect of patients [14–16].

The results of our study show that individuals carrying the *OPRM1* 118A allele are more sensitive to pain. Single-factor analysis of the VAS showed that carriers of A gene with *OPRM1* A118G polymorphism were risk factors for VAS greater than 4. Further regression analysis revealed that the occurrence probability of GA and AA type subjects with VAS ≥ 4 was 13.73 times high than that of GG homozygous subjects; the adjusted dose–effect relationship diagram (Fig. 2) clearly shows that after the age, sex, weight, height, fentanyl dosage during operation, operation duration, and *CYP3A4* *1G and *CYP3A5* *3 polymorphisms are adjusted, the possibility of PACU VAS ≥ 4 in patients with A gene was significantly increased. This is consistent with previous clinical studies that found patients with the A gene had higher VAS pain scores and a greater need for exogenous opioids post-surgery [13]. The endogenous and exogenous analgesic effects mediated by opioid receptors are mainly regulated by their genes. The common locus of *OPRM1* gene polymorphism is A118G, which is located in exon 1. This mutation leads to a substitution of G for A. This substitution results in a change in the expression of the 40th amino acid in the extracellular N-terminal region of the μ-opioid receptor, from aspartic acid to asparaginase, contributing to the loss of a glycosylation site in this region. As a result, the mutant receptor binds β-endorphin more strongly [18], affecting its metabolism and utilization.

In addition, in our study, we also found that the amount of fentanyl used by A gene carriers in PACU was significantly higher than that of GG patients. This may be due to the phenotypic effect of the substitution of guanylate G for adenylate A in A118G homozygous mutation patients, which further enhances the endogenous opioid system activity of mutant individuals, thereby reducing their sense of pain [17]. Molecular-level research [18, 19] also shows that the activity of the endogenous opioid system of mutant individuals is enhanced, as is the effect of endogenous opioid substances. In the future clinical practice, it is expected to screen out allele carriers who are more sensitive to pain but have poor effects on exogenous drugs through preoperative gene testing, and give them more appropriate anesthetic treatment schemes.

## Limitations

The limitations of the study include the fact that the genes markers currently included in the study are not comprehensive enough. In recent years, to predict the effect of gene polymorphism on anesthesia safety, other studies have reported that gene polymorphism such as CYP3A4*1G and *ABCB1* C3435T is related to the dose of fentanyl and anesthesia tolerance [20]. On the other hand, due to the limited size of the study cohort, we were unable to create an independent validation set to assess the performance of research in the development dataset. Our study was based on retrospective clinical data, and the characteristics of subjects are clinical basic data and routine examination results; thus, other novel markers are not included.

Postoperative complications are also closely related to pain management [21, 22]. The study of Boström P et al. [21] revealed that anastomotic leakage is an independent marker of increased patients’ pain in the PACU. Our study only focused on the short-term prognosis, and the postoperative follow-up was limited to 48 h. Moreover, long-term factors such as anastomotic leakage, TNM staging, metastasis, and recurrence were not included in the basic characteristics of patients for analysis. These limitations undermine the clinical value and applicability of this paper to a certain extent. In the future research, we will focus on the correlation between postoperative pain degree and more clinical parameters, complications, as well as the incidence of adverse reactions.

Besides, the small sample size (101 patients) of our study may affect the decision about which and how many factors were included in the adjusted analysis. And the stability and applicability of the adjusted model needs to be verified and evaluated in future cohort studies with larger sample sizes.

## Conclusion

Our findings revealed that carrying the A gene of *OPRM1* was highly associated with an increase in fentanyl dose and VAS ≥ 4 in the PACU. It is important for the clinical to pay attention to gene detection in anesthetic drugs before the operation. It has provided a novel way to analyze the influencing factors of VAS, and may be the valuable clinical significance for selecting a suitable anesthetic scheme.

## Supplementary Information

**Additional file 1.** 

## Data Availability

The datasets generated and/or analysed during the current study are available in the Genome Variation Map (GVM) repository, [https://bigd.big.ac.cn/gvm/getProjectDetail?Project=GVM000503].
