# Supplementary material for: Is OPRM1 genotype a valuable predictor of VAS in patients undergoing laparoscopic radical resection of colorectal cancer with fentanyl?
Source: BMC Anesthesiol. 2023 May 22;23:173. doi: 10.1186/s12871-023-02120-1 (PMC10201726; doi:10.1186/s12871-023-02120-1)
Supplement: Supplementary file 1 — Additional file 1. [file 12871_2023_2120_MOESM1_ESM.docx]

Supplementary Table1. Characteristics of subjects in the research

| Characteristic | VAS<4 | VAS≥4 |
| --- | --- | --- |
| NO of paticipants | 92 | 9 |
| Diameter of tumor | 3.99 ± 1.96 | 3.48 ± 2.12 |
| Comorbidity |  |  |
| 0 | 66 (71.74%) | 6 (66.67%) |
| 1 | 26 (28.26%) | 3 (33.33%) |
| ASA |  |  |
| I | 4 (4.35%) | 2 (22.22%) |
| II | 88 (95.65%) | 7 (77.78%) |
| Tumor location |  |  |
| right | 57 (61.96%) | 4 (44.44%) |
| left | 35 (38.04%) | 5 (55.56%) |
